# Label-free Chemical Imaging of Fungal Spore Walls by Raman Microscopy and Multivariate Curve Resolution Analysis

**DOI:** 10.1038/srep27789

**Published:** 2016-06-09

**Authors:** Hemanth Noothalapati, Takahiro Sasaki, Tomohiro Kaino, Makoto Kawamukai, Masahiro Ando, Hiro-o Hamaguchi, Tatsuyuki Yamamoto

**Affiliations:** 1 Raman Center for Medical and Biological Applications, Shimane University, Matsue 690-8504, Japan; 2 Faculty of Life and Environmental Science, Shimane University, Matsue 690-8504, Japan; 3 Consolidated Research Institute for Advanced Science and Medical Care, Waseda University, Tokyo 162-0041, Japan; 4 Institute of Molecular Science and Department of Applied Chemistry, National Chiao Tung University, Hsinchu 30010, Taiwan

## Abstract

Fungal cell walls are medically important since they represent a drug target site for antifungal medication. So far there is no method to directly visualize structurally similar cell wall components such as α-glucan, β-glucan and mannan with high specificity, especially in a label-free manner. In this study, we have developed a Raman spectroscopy based molecular imaging method and combined multivariate curve resolution analysis to enable detection and visualization of multiple polysaccharide components simultaneously at the single cell level. Our results show that vegetative cell and ascus walls are made up of both α- and β-glucans while spore wall is exclusively made of α-glucan. Co-localization studies reveal the absence of mannans in ascus wall but are distributed primarily in spores. Such detailed picture is believed to further enhance our understanding of the dynamic spore wall architecture, eventually leading to advancements in drug discovery and development in the near future.

Spores are quiescent forms of microbial life that preserve the genetic material when conditions are lethal for normal vegetative life cycle. *Schizosaccharomyces pombe* (*S. pombe*) is a rod shaped, single-celled haploid organism that generally reproduces asexually by mitosis. However, nutrient deprivation, especially that of nitrogen, leads to a series of complex biochemical, genetic and morphological changes that triggers cells to exit the mitotic cycle and enter sexual reproductive cycle where a diploid is formed by mating with the opposite strain, followed by sporogenesis. Sporulation is a dynamic process that involves segregation of chromosomes in two consecutive meiotic divisions, a form of cell division that is radically different from the vegetative cells, resulting in the production of four haploid nuclei. These are first enveloped within double structured prospore membrane in the cytoplasm of the mother cell followed by spore wall formation, which is rather extensive when compared with their vegetative counterparts^1^,^2^,^3^,^4^. In the case of *S. pombe*, haploid spores are formed in a set of four, called a tetrad. Spore containing sac is called an ascus while the overall structure is termed an ascospore. Spore walls, which are usually made up of complex three-dimensional network of polysaccharides such as glucan, mannan, chitin, chitosan and glycoproteins, confer mechanical strength and resistance to environmental stress. In fact, yeast spores are known to enable them to survive passage through the digestive tract of *Drosophila* and are shown to be resistant to laboratory treatments such as exposure to ether vapor, temperature & pH shock and very high salt concentrations^5^,^6^,^7^.

From a pathological perspective, cell wall acts as the first line of defense against the host in the event of fungal infection. Since most of the wall components are unique to fungi but absent in mammalian cells, it serves as one of the attractive targets for anti-fungal drug development^8^,^9^. Despite the advances in drug discovery and therapy, fungal infections are ever increasing and remain a significant problem, especially in immunosuppressed hosts (transplant recipients, cancer and AIDS patients), as spores of opportunistic human fungal pathogens like *Candida spp and Cryptococcus spp* are prevalent everywhere in our environment^10^,^11^,^12^. Indeed, recent studies have shown that fungal spores and other biological particles account for a large proportion aerosol particle mass from both rural and urban areas^13^. Though yeasts are one of the extensively used eukaryotic models in biology, their cell and spore walls are incompletely understood due primarily to the lack of methods that can let us visualize different polysaccharide components simultaneously. In fact, *Saccharomyces cerevisiae* and *Aspergillus fumigatus* are the only two fungi whose vegetative cell wall have been investigated in detail^8^. Most of the information on cell walls currently available was either obtained using electron microscopy, which not only requires fixing but also lacks chemical specificity, or genetic and biochemical methods where cell fractionation, isolation and purification procedures were employed^4^,^14^,^15^,^16^. Since it was technically not possible to analyze cell wall polymers without prior enzymatic or physicochemical treatment of the wall, researchers started using fluorescence based labeling techniques to observe some spore wall components^17^. But, distinguishing, for example, α- and β-glucans in the mixture becomes next to impossible as they only differ in their configuration at the anomeric carbon. As none of the aforementioned methods give a complete picture of cell wall architecture, we set out to develop a Raman spectroscopy based molecular imaging method to identify and visualize various cell wall components of yeast cells and spores. Raman spectrum, otherwise called a molecular fingerprint, provides rich chemical information with high specificity. Also, Raman scattering, a result of inherent molecular vibrations, requires no exogenous probe. Therefore, Raman microscopy based method enables *in vivo* label-free molecular detection with subcellular resolution^18^,^19^.

*S. pombe* whose cell wall primarily consists of branched β-(1,3)-glucans (45–55%), linear α-(1,3)-glucans (18–25%), α-galactomannan (9–14%) was chosen for this study^14^,^20^. One of the most interesting aspects during sporulation in *S. pombe* is that, unlike mitosis where cell wall is extended from the old cell, spore wall is made from the scratch *de novo* and assembled within^1^. Hence, spore wall could very well be structurally different from vegetative cells’ and calls for a deeper understanding of the wall architecture, for it is both interesting and fundamentally important with far reaching implications in biology and medicine.

## Results

### Space-resolved Raman Spectra of Fission Yeast Cells and Spores

**Figure 1:**
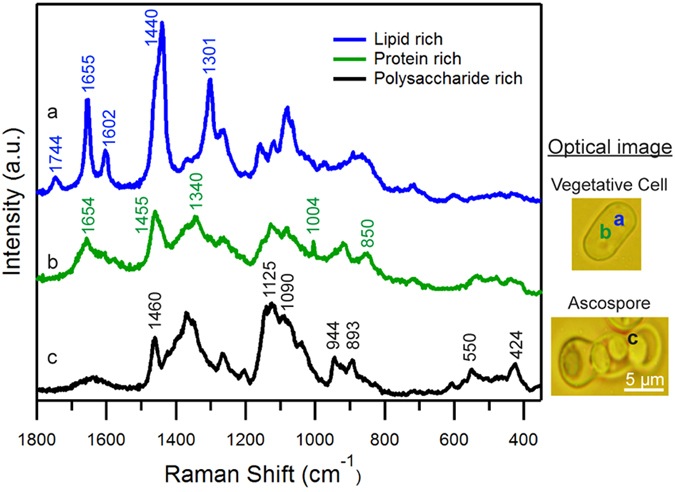
Space-resolved Raman spectra of a single living fission yeast vegetative cell and spore. Spectra (**a**,**b**) were obtained from lipid droplet and cytoplasmic region, respectively, from a vegetative cell while spectrum (**c**) was measured from a region close to ascus and spore wall (designated as yeast wall spectrum). Corresponding optical images are included and the measured points are indicated using alphabets.

To obtain a holistic view of biomolecules present, space-resolved Raman spectra were measured from three distinct regions namely lipid droplets, cytoplasm and cell wall in both spores and vegetative cells. Raman spectrum representative of each intracellular location is shown in Fig. 1. Major features in lipid Raman spectrum (Fig. 1a) include C=O stretch of ester linkage at 1744 cm^−1^, C=C stretch at 1655 cm^−1^, C=C diene in-phase stretch of ergosterol at 1602 cm^−1 ^21, C-H bend of the aliphatic chain at 1440 cm^−1^ and in-plane CH_2_ twist at 1301 cm^−1^. Spectra measured from cytoplasm (Fig. 1b), which usually contains high concentrations of dissolved macromolecules such as proteins, showed Raman bands of amide I at 1654 cm^−1^, C-H bend at 1455 cm^−1^ and 1340 cm^−1^, ring breathing modes of phenylalanine at 1004 cm^−1^ and tyrosine band in 850 cm^−1^ region^22^. Due to the absence of lipid markers such as 1744 cm^−1^ and 1301 cm^−1^, it is clear that contribution from lipids is negligible in protein rich spectrum. It is important to note that Raman bands in spore wall spectrum (Fig. 1c) include COO^−^ asymmetric stretch at 1637 cm^−1^, COO^−^ symmetric stretch and C-H deformation modes between 1500–1200 cm^−1^, C-C stretch and C-O-C glycosidic link and symmetric ring breathing modes between 1200–1000 cm^−1^, side group vibrations between 1000–800 cm^−1^ and δ(CCC) ring deformation modes below 460 cm^−1^, indicating mixture of various polysaccharides^23^.

### Identification of Raman marker bands specific to α- and β- glucans

**Figure 2:**
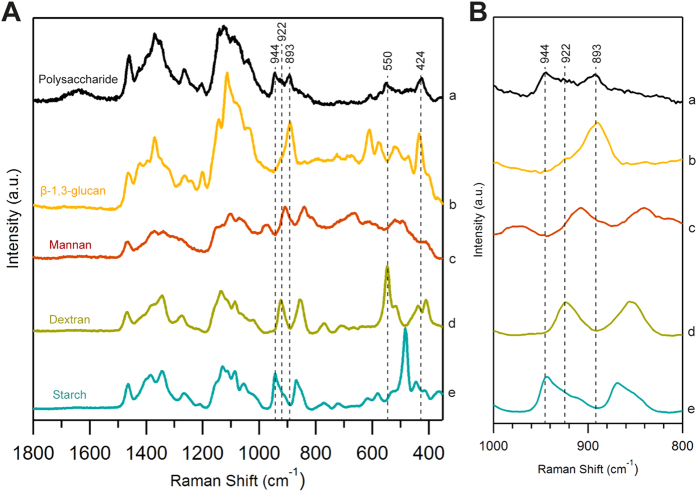
Comparison of yeast wall Raman spectrum with standard polysaccharides. (**A**) Whole fingerprint region and (**B**) zoom up of glucan marker region (1000–800 cm^−1^). (a) Yeast wall spectrum [same as **c** in Fig. 1], (b) β-(1,3)-glucan, (c) mannan, (d) dextran and (e) starch.

As discussed earlier, glucan is the most abundant (~60–70%) structural biopolymer in fission yeast cell walls. It is mainly composed of β-(1,3)-glucan (with occasional β-(1,6)-glucan branches), α-glucan and the next major component being α-galactomannan. So, in order to identify marker bands specific to each major component, we compared spore wall spectrum with standard polysaccharide candidates – β-(1,3)-glucan, dextran, starch and mannan (Fig. 2). In the present case, bands only below 1000 cm^−1^ were used for identification purpose, as other bands provide no specific information.

Both α- and β-glucan are polysaccharides of glucose monomers which differs only in the configuration at the anomeric C1 position. Raman spectrum in the region between 900–800 cm^−1^ is sensitive to glycosidic linkages. Studies on series of carbohydrate monomers showed C-H equatorial bending vibrations for β- anomer between 905–885 cm^−1^ and for α- type in between 865–835 cm^-1 ^24,25. It is important to note that the polysaccharide rich spectrum was not obtained from a pure chemical but a single spore that also contains many other bio-macromolecules whose Raman spectrum may have overlapping bands in this region. Careful examination of space-resolved spectra reveals spectral window in the region utilized for detecting β form while bands from tyrosine residues (850 cm^−1^ region) in protein rich spectrum interferes in α-glucan detection. Hence β form can be readily identified by comparing spore wall spectrum and pure β-(1,3)-glucan. Raman band at 893 cm^−1^ that coincides very well, serves as a β-glucan marker. Since the broad feature at ~850 cm^−1^ does not give any information about the presence of α-forms in this scenario, we screened for other Raman markers that can let us distinguish α-glucans. Dextran which contains primarily α-(1,6)-glucan with α-(1,3) branches and starch α-(1,4)-glucan with α-(1,6) branches were selected for identifying bands specific to α-glucans. As expected, both of them lack 893 cm^−1^ Raman band but have bands in α- anomeric region. In addition to that, they have bands in between 960–900 cm^−1^ corresponding to asymmetrical ring vibrations and between 600–460 cm^−1^ from δ(COC) glycosidic linkage. Dextran shows ring vibrations at 922 cm^−1^ and glycosidic band at 550 cm^−1^ while starch shows ring vibrations at 944 cm^−1^, all of which perfectly overlap with yeast wall spectrum. Moreover, our identification corroborates well with available literature as these bands were also previously assigned to α-glucans^26^,^27^. Since the band at 922 cm^−1^ is broad and appears as a shoulder overlapping with 944 cm^−1^ only the other two bands (550 cm^−1^ and 944 cm^−1^) can be used as markers to identify α-glucan component of the fungal cell wall. Raman band at 424 cm^−1^ (region dominated by skeletal vibrations) can be used as a non-specific glucan marker as it can be observed in all of these spectra. Lastly, no specific bands were observed in a thorough comparison of polysaccharide rich spore wall spectrum and mannan leaving it undetectable by univariate analysis. Thus α- and β-glucan components of the cell wall can be distinguished by Raman microspectroscopy in a straightforward manner while we believe information about other important structural component, mannan, can be extracted by applying multivariate statistics.

### Visualization of glucan component of yeast cell wall and spore wall by univariate Raman imaging

**Figure 3:**
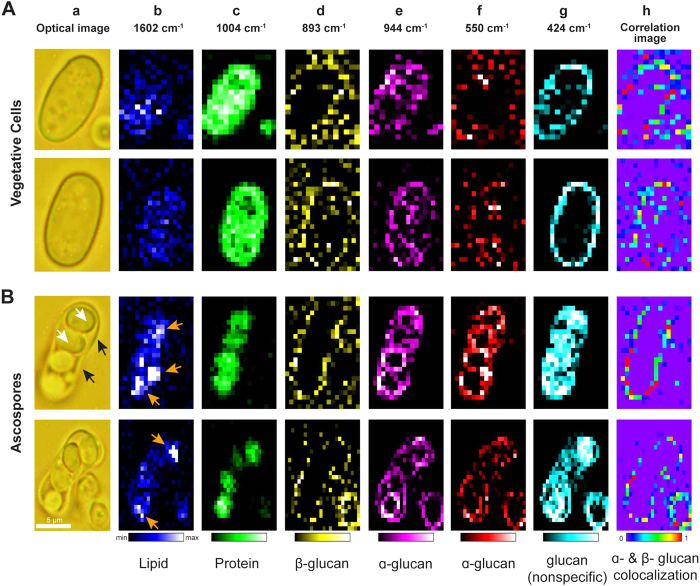
Univariate Raman imaging of (**A**) *S. pombe* vegetative cells and (**B**) spores. (a) Bright field optical images. Scale bar measures 5 μm. White arrows indicate spores and black arrows indicate ascus wall. (b–f) Raman images constructed for (b) lipids using 1602 cm^−1^ [gold arrows indicate lipid accumulation], (c) proteins using 1004 cm^−1^, (d) β-glucans using 893 cm^−1^, (e) α-glucans using 944 cm^−1^, (f) using 550 cm^−1^ and (g) both glucan anomers using skeletal vibrations at 424 cm^−1^. (h) Correlation images obtained using equation 1 between (d) & (e) shows colocalization of α- and β-glucans only in the ascus.

After having gained insights into Raman markers specific to α- and β-glucans, we tried to obtain their distribution images in both vegetative cells and spores (Fig. 3) by mapping the intensity of these individual bands. Initially, major components such as lipids and proteins were mapped to get an overall idea of intracellular activity. Raman images constructed using 1602 cm^−1^ from ergosterol (Fig. 3b) revealed accumulation of lipid droplets in spores while it is more or less evenly distributed in vegetative cells. This could be the result of storage of energy in the form of lipid droplets under stress, which will be used when reentering vegetative phase under favorable conditions. Similarly, maps obtained using Phe ring breathing vibration at 1004 cm^−1^ (Fig. 3c) revealed much lower protein activity in spores compared to normal cells. This is understandable because spores, also called as dormant cells, are known to have low metabolic activity than their normal counterparts.

Cell wall structure of fission yeast has been extensively studied but the actual structure of spore wall is not well understood in *S. pombe*. So, we first looked into β-glucan component whose Raman images were constructed using 893 cm^−1^ band (Fig. 3d). In vegetative cells, its distribution could be seen at the cell periphery as expected, indicating the cell wall region. But, in the case of ascospores, surprisingly it was distributed only in the mother cell wall (ascus wall) but not in individual spore wall. On the other hand, α-glucan images obtained from two marker bands, 944 cm^−1^ (Fig. 3e) and 550 cm^−1^ (Fig. 3f), revealed distribution patterns mainly in the spores, in addition to its presence in mother cell wall. However, α-glucan being a minor component in vegetative cell wall, understandably showed weaker distribution when compared to spores. Raman images from 424 cm^−1^ (Fig. 3g) a nonspecific marker representing glucan in general, show clear distribution in both vegetative cell and spore wall. In fact, it is relatively more intense in spore wall than ascus wall due to the greater contribution from α-glucans. In order to further our understanding of the fungal wall and to examine regions where both anomers are colocalized, we performed structural cross–correlation image analysis for Raman images of β-glucan (Fig. 3d) and α-glucan (Fig. 3e). Correlation coefficient *C*_*ij*_ was calculated using equation 1,


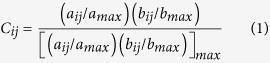


where ***a***_***ij***_ and ***b***_***ij***_ represent intensities at ***(i,j)***th pixel of the two images ***a*** and ***b***^22^. The correlation image analysis where signal intensities are compared on a pixel-by-pixel basis (Fig. 3h) showed strong correlation in ascus wall but not in spore wall within the ascospore.

β-glucan which was found to be an essential component for maturation of ascospore wall and spore viability in *S. pombe*^28^, indirectly led researchers to believe in a similar wall structure for vegetative cells and spores but, our imaging results suggest otherwise. Though β-glucans are generally more abundant than α-glucans in normal vegetative cells (58% β versus 28% α), Sanchez and co-workers noted that the situation was opposite in *S. pombe* spores (38% β versus 46% α)^29^. However, one important point to take into consideration is that their study employed cell fractionation procedures to isolate cell wall in estimating glucan anomers. Such an experiment can only provide cell-averaged data in the form of ratios of β/α glucan present in vegetative cells and ascospores but not spore specific data or its distribution in spore wall. Our imaging result supports the view that estimation of ascospore β-glucans in their study should have been solely contributed by ascus wall component. Thus we could arrive at a conclusion that while α- glucans are present in both ascus and spore wall, β-glucans are distributed only in ascus wall.

### Extracting mannan component and its distribution by multivariate data analysis

**Figure 4:**
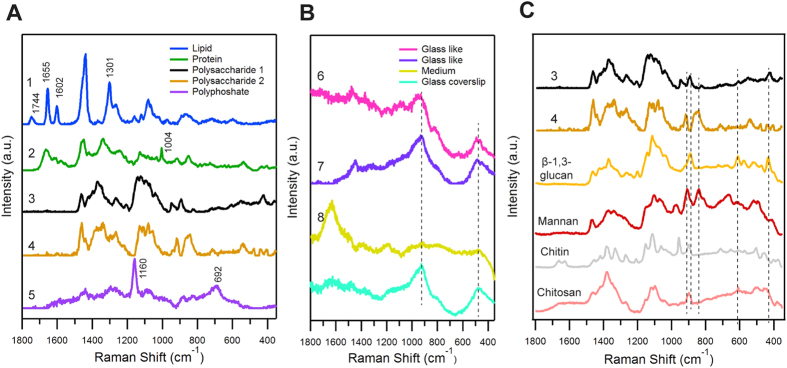
Results of MCR analyzed with eight components. (**A**) MCR spectral components 1-5, (**B**) components 6–8 in comparison with Raman spectrum measured from glass coverslip, (**C**) Components 3 and 4 (same as in (**A**) compared with standard polysaccharide Raman spectra; β-(1,3)-glucan (gold), mannan (maroon), chitin (grey) and chitosan (light red).

In the case where required information is not obtainable using a single band, multivariate data analyses such as principal component analysis (PCA), cluster analysis and multivariate curve resolution (MCR) remain a preferred approach. Multivariate methods involve global analysis of spectral data where observations are made for more than one variable at a time. PCA gives an orthogonal set of principal spectral components that have both positive and negative values. Physical meaning of these decomposed spectra is not clear, as they do not represent any pure component. In order to obtain insights into mannan distribution, the latter two methods seem appropriate^30^,^31^,^32^,^33^. Previously, we have developed and applied MCR to understand lipid metabolism *in vivo*, in both qualitative and quantitative manner, at the single cell level^21^,^34^. In this work, by employing MCR, a matrix factorization method, to our Raman hyperspectral data (a large matrix obtained by combining data from each individual cell), we successfully separated eight components (Fig. 4) that represent the whole data very well.

**Figure 5:**
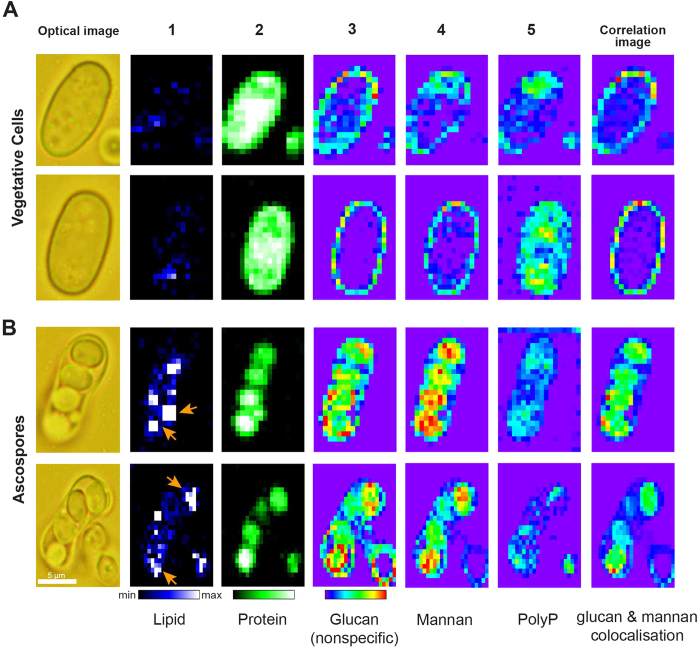
Raman images constructed from MCR analysis: (**A**) *S. pombe* vegetative cells and (**B**) ascospores. MCR component distributions images from of (1) Lipid [gold arrows indicate lipid accumulation], (2) protein, (3) glucans, (4) mannan, (5) polyphosphates (PolyP) and correlation profiles between glucan and mannan showing colocalization in spores. Corresponding bright field optical images are included for reference.

**Figure 6:**
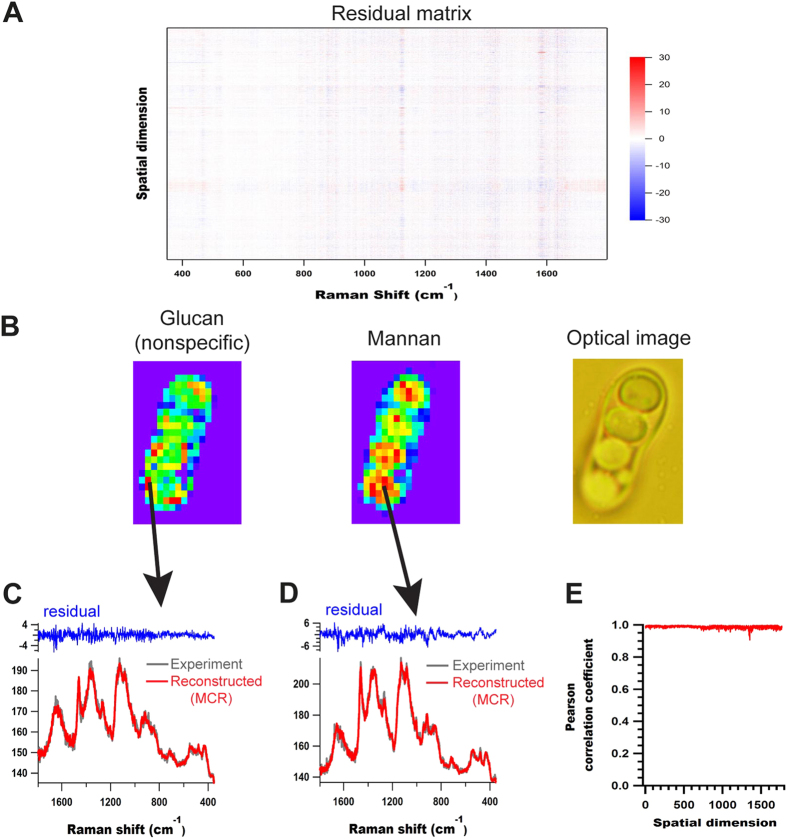
MCR residual analysis. (**A**) Residual matrix, (**B**) Optical image of *S. pombe* vegetative cell with MCR images from 3rd and 4th components - glucan and mannan respectively- [same as Fig. 5], (**C**,**D**) comparison of experimental and MCR reconstructed data along with corresponding residuals from two representative points rich in glucan and mannan respectively, as indicated by black arrows and (**E**) Pearson correlation coefficients obtained for each point before and after MCR.

Spectrum 1 (Fig. 4A-1) with bands at 1744 cm^−1^, 1655 cm^−1^, 1602 cm^−1^, and 1301 cm^−1^, which also appear similar to lipid rich spectrum obtained during space-resolved experiment, represents lipid component. Spectral pattern of 2 (Fig. 4A-2) with bands at 1654 cm^−1^ (amide I) and 1004 cm^−1^ (Phe) indicates protein component. Once again, absence of 1744 cm^−1^ and 1301 cm^−1^ bands in spectrum 2 signifies good separation of lipid and protein components. Spectra 3 and 4 (Fig. 4A-3), which look similar to yeast wall spectrum, represent two polysaccharide components and will be discussed in detail. Component 5 (Fig. 4A-5), which has bands at 1160 cm^−1^ and 692 cm^−1^ correspond to PO_2_^−^ and P-O-P stretch, respectively, indicates the presence of inorganic polyphosphates (PolyP). The next two components, namely 6, 7 (Fig. 4B-6) are contributions from glass substrate in which contribution from fluorescence background differs. Blank spectrum obtained from glass coverslip is also compared in the same panel. The final spectrum 8 (Fig. 4B-8) with OH bending vibrational band indicates water/medium component.

In order to understand molecular origin of components 3 and 4 from MCR analysis, Raman spectra from a series of potential polysaccharide candidates were measured and compared (Fig. 4C). By a way of simple comparison of the whole spectrum, it can be easily understood that neither 3 nor 4 resembles chitin or chitosan. Though the other two components look similar, a thorough look into bands especially below 1000 cm^−1^ reveal that component 3 is more close to pure β-(1,3)-glucan with bands at 893 cm^−1^, and 424 cm^−1^ overlapping perfectly. In addition to these bands, 944 cm^−1^ and 550 cm^−1^ also appear which are from α-glucans. However, α- and β-glucans could not be further separated individually by increasing the number of components, for their spectral similarity is very high. Hence component 3 can serve as a nonspecific glucan marker similar to 424 cm^−1^ band from univariate analysis. As one would expect, they are absent in 4 indicating perfect separation of each component in our analysis. A careful comparison, especially in the anomeric spectral region (900–800 cm^−1^), revealed component 4 to be mannan.

Once molecular assignments are established, corresponding MCR Raman images were constructed to visualize their intracellular distribution (Fig. 5). First, lipid component (Fig. 5-1) showed accumulation in spores but not in vegetative cells. Protein component (Fig. 5-2) showed much lower intensity distribution in spores compared to vegetative cells indicating reduced metabolic activity in spores. Glucan images (Fig. 5-3), which represent α- and β-glucans together, showed intense distribution pattern in spore wall compared to ascus wall and vegetative cell wall (similar to 424 cm^−1^).

All three trends agree very well with results from univariate analysis done above (Fig. 3-a,b,g respectively). These results further validate the separation of pure chemical components by MCR to be successful and complete. Next, we looked into mannan images from MCR analysis (Fig. 5-4). In vegetative cells, it showed distribution near the edges, similar to glucan component, indicating cell wall. In the case of ascospores, it is scarcely distributed in ascus wall compared to spore wall. Even more surprising is the fact that it is more intense in the region seemingly inside spores than spore wall. Depth resolution in this experiment (~2.5 μm) would not allow further verification as to whether mannan is distributed truly inside the spore or its surface. It is known that inner layers of budding yeasts spores are predominantly composed of mannan while the outer layers are made of glucan. Our imaging results also suggest that glucan layer encompasses mannan rich structures in fission yeast spores. PolyP images (Fig. 5) reveal intracellular locations where phosphates are present. PolyP, depending on its location, has diverse roles ranging from phosphate storage in cytosol to regulation of gene expression in nuclei. They are also known to be present in yeast vacuoles, mitochondria and plasma membranes under normal vegetative conditions^35^. Quite different localization of PolyP in the two seemingly similar vegetative cells might be the result of different origins of phosphates. It suggests that the role of PolyP might be as well different reflecting cellular individuality. When it comes to ascospores, the presence of phosphates might be accounted for the signaling nucleotides that are heavily phosphorylated, marking the development of ascospore under nutrient deprived conditions^36^. To gain further insights in to the cell wall architecture, correlation images between glucan and mannan were constructed. Under vegetative conditions, colocalization of glucan and mannan was seen only in the cell wall where as in ascospores, these biopolymers were observed primarily in spores and spore wall but not in ascus wall [refer to Supplementary Fig. 3 for details pertaining to glass and background component images].

In order to estimate the robustness of MCR analysis and to estimate fitting errors, residual matrix was constructed as shown in Fig. 6A. It contained low and random noise with no abnormal features. We then compared experimental data with MCR reconstructed ones at representative points rich in glucan and mannan (Fig. 6C,D). Further Pearson correlation coefficient, between original data and MCR fit at each point, was calculated as a measure of similarity and was found to be very high with an average value of 0.99 (Fig. 6E). Thus it is clear that MCR fitting is complete and the data is well reconstructed. Additionally, we also carried out agglomerative hierarchical clustering analysis (AHCA), another popular unsupervised multivariate method to separate spectral components in living cells, to study glucan and mannan distributions and to compare the efficacy of our MCR analysis. Indeed, AHCA failed to separate individual biopolymer components [refer to Supplementary Fig. 4].

## Discussion

In this work, we have developed a Raman microspectroscopic method to obtain detailed information about complex fungal vegetative cell and spore wall architectures without needing any fluorescent labels and/or electron microscopes. We show that by focusing on glucan specific bands in a univariate approach, both α- and β- anomers of glucan can be readily identified in a simple and straightforward manner. While there is no significant difference in distribution of α- and β-forms in vegetative cells, they colocalized only in the ascus or mother cell wall of the ascospore but not in spore walls. In contrast β-glucan was not detected in spore wall, which, at this stage of the cell cycle, is predominantly made up of its α-anomer. Since β-glucan form the major biopolymer component in vegetative cells’ wall, it is not only surprising but also intriguing as to how morphogenetic changes occur when spores exit their passive lives and enter mitotic cycle under favorable conditions.

We further show that Raman imaging experiment, when combined with MCR, not only yields information about major biomolecules such as lipids, protein and glucans but also about minor mannan component which is otherwise not possible to detect without genetic manipulations. Results from MCR analysis are highly desirable as it separates pure chemical spectra that are meaningful. Though Raman imaging in itself gives information on relative abundance of these components in a cell, we must however understand that absolute quantification is difficult. Additionally, without any prior information on phosphates, PolyP was separated highlighting the fact that such a method could be highly productive in exploratory studies. Cell wall, now being recognized as a dynamic organelle, not only differs depending on the fungal species, but is also influenced by external environmental factors. The very possibility of studying fungal cell and spore walls in such detail, which was previously not possible, opens up new directions in biomedical research. In fact, efforts to understand the effects of anti-fungal drugs that target cell walls are underway in our laboratory. Another example where we envisage application of Raman spectroscopy is in the detection of glucan and mannan markers for early diagnosis of life threatening invasive fungal infections, which is the need of the hour in case of aspergillosis and in patients with hematological malignancies^37^,^38^,^39^,^40^. We believe Raman microscopy in combination with biochemical methods will serve a powerful tool in both clinical applications for non-invasive disease diagnostics and fundamental studies to unravel yet unknown roles of these structural biopolymers and the mysteries surrounding the cell wall architecture.

## Methods

### Sample

A wild type *Schizosaccharomyces* strain L968 with h90 genotype was used in this study. Rich YES medium containing yeast extract (5 g/l) and glucose (3 g/l) with additional supplements was used for pre-culture. Solid medium was prepared by adding agar (20 g/l). Vegetative cells were cultured for 3 days in PM+N [potassium hydrogen phthalate −3 g/l, Na2HPO4 −2.2 g/l, NH4Cl −5 g/l, glucose −20 g/l, salts, vitamins and minerals] while sporulation was done in PM-N medium, which is similar to the former but lacks NH4Cl. For Raman measurements, cells were harvested after said time, washed twice with water, put in a glass bottom dish and transferred directly to our Raman microscope without further sample treatment.

### Confocal Raman Microscopy

Raman microspectroscopic and imaging experiments were done with a homemade confocal Raman microscope [refer to Supplementary Fig. 1 for optical layout]. Briefly, the 632.8 nm output of a He-Ne laser was used as the Raman excitation source. The laser beam was first expanded, introduced to an inverted microscope (Olympus, IX70) and tightly focused on to the sample with a high magnification oil immersion objective lens (100×, NA = 1.3). Backscattered light collected by the same objective lens is passed through an edge filter to block Rayleigh light and then through a 50 μm confocal pinhole before being introduced into an imaging spectrometer (Chromex, 250IS) and detected by a back illuminated, liquid nitrogen cooled CCD detector (Princeton Instruments, Spec-10). Raman experiments were done using slit width of 50 μm and a grating with 600 gr/mm. The aforementioned conditions determine spectral resolution to be 0.25 nm, which translates to ~4.5 cm^−1^. Laser power at the sample point was 5 mW. An exposure time of 60 s was used for space-resolved measurements while just 1 s/pixel was used during imaging experiments. The lateral and axial resolutions achieved were 300 nm and 3 μm, respectively. All measurements were done at room temperature (24 °C).

### Multivariate Analysis

(a) *Multivariate Curve Resolution Analysis*. Imaging data from experiment is in the form of a non-negative matrix, say **A**, of dimensions *m* × *n* (*m* denotes number of points per spectrum and *n* the total number of spectra). In MCR analysis, matrix approximation sought by a linear combination of desired number of spectral components can be written as


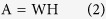


In this low-rank approximation, columns of **W** (an *m* × *k* matrix) represent pure component spectra and rows of **H** (a *k* × *n* matrix) represent intensity profile of each spectral component. The parameter *k*, the number of components, was set to 8 in this study as estimated from singular value decomposition (SVD) analysis [refer to Supplementary Fig. 2 for analyses with *k* = 7 and *k* = 9 showing failure in separation]. **W** and **H** were iteratively refined using alternating least squares so that the Frobenius norm ||**A-WH**||^2^ is minimized with non-negative constraints **W **≥ 0 and **H **≥ 0. To obtain sparser solutions, additional L _1_ penalty term (lasso regression) of *α*^*2*^ = 0.00025 was applied as


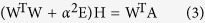



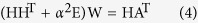


where **E** is a *k* × *k* matrix whose elements are all unity. Additionally L_2_ penalty term (ridge regression) of *β*^*2*^ = 0.00025 was also applied as follows.


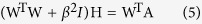



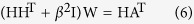


where **I** is a *k* × *k* identity matrix.

Residual matrix (A-WH) was constructed to estimate fitting error. MCR analysis was performed on software specifically developed for Raman spectral decomposition (nmf-11, Pylone).

*(b) Cluster Analysis*. AHCA was performed using hyperSpec package in R, version 3.1.2. Euclidean distance measure was used to calculate distance matrix. Dendrogram was obtained by employing Ward’s algorithm to merge spectra with high similarity into clusters.

## Additional Information

**How to cite this article**: Noothalapati, H. *et al*. Label-free Chemical Imaging of Fungal Spore Walls by Raman Microscopy and Multivariate Curve Resolution Analysis. *Sci. Rep*. **6**, 27789; doi: 10.1038/srep27789 (2016).

## Supplementary Material

Supplementary Information

## References

[b1] Aaron M. N. Sporulation in the Budding Yeast Saccharomyces cerevisiae. Genetics 189, 737–765 (2011). 22084423 10.1534/genetics.111.127126 PMC3213374

[b2] Neiman A. M. Ascospore formation in the yeast Saccharomyces cerevisiae. Microbiol Mol Biol Rev 69, 565–584 (2005). 16339736 10.1128/MMBR.69.4.565-584.2005 PMC1306807

[b3] Yoo B. Y., Calleja G. B. & Johnson B. F. Ultrastructural changes of the fission yeast (Schizosaccharomyces pombe) during ascospore formation. Arch Mikrobiol 91, 1–10 (1973). 4711455 10.1007/BF00409533

[b4] Tanaka K. & Hirata A. Ascospore development in the fission yeasts Schizosaccharomyces pombe and S. japonicus. J Cell Sci 56, 263–279 (1982). 7166567 10.1242/jcs.56.1.263

[b5] Dawes I. & Hardie I. Selective killing of vegetative cells in sporulated yeast cultures by exposure to diethyl ether. Molec Gen Genet 131, 281–289 (1974). 4612332 10.1007/BF00264859

[b6] Briza P., Breitenbach M., Ellinger A. & Segall J. Isolation of two developmentally regulated genes involved in spore wall maturation in Saccharomyces cerevisiae. Genes & Development 4, 1775–1789 (1990). 2249774 10.1101/gad.4.10.1775

[b7] Coluccio A. E., Rodriguez R. K., Kernan M. J. & Neiman A. M. The yeast spore wall enables spores to survive passage through the digestive tract of Drosophila. PLoS One 3, e2873 (2008). 18682732 10.1371/journal.pone.0002873 PMC2478712

[b8] Latge J. P. The cell wall: a carbohydrate armour for the fungal cell. Mol Microbiol 66, 279–290 (2007). 17854405 10.1111/j.1365-2958.2007.05872.x

[b9] Carrillo-Munoz A. J., Giusiano G., Ezkurra P. A. & Quindos G. Antifungal agents: mode of action in yeast cells. Rev Esp Quimioter 19, 130–139 (2006). 16964330

[b10] Velagapudi R., Hsueh Y. P., Geunes-Boyer S., Wright J. R. & Heitman J. Spores as infectious propagules of Cryptococcus neoformans. Infect Immun 77, 4345–4355 (2009). 19620339 10.1128/IAI.00542-09 PMC2747963

[b11] Arielle B., Damian J. K. & William E. G. Antifungal Drug Discovery: Something Old and Something New. PLoS Pathogens 8, (2012). 10.1371/journal.ppat.1002870 PMC3435257 22969422

[b12] Aperis G., Myriounis N., Spanakis E. K. & Mylonakis E. Developments in the treatment of candidiasis: more choices and new challenges. Expert Opin Investig Drugs 15, 1319–1336 (2006). 10.1517/13543784.15.11.1319 17040194

[b13] Frohlich-Nowoisky J., Pickersgill D. A., Despres V. R. & Poschl U. High diversity of fungi in air particulate matter. Proc Natl Acad Sci USA 106, 12814–12819 (2009). 19617562 10.1073/pnas.0811003106 PMC2722276

[b14] Kopecka M., Fleet G. H. & Phaff H. J. Ultrastructure of the Cell-Wall of Schizosaccharomyces-Pombe Following Treatment with Various Glucanases. Journal of Structural Biology 114, 140–152 (1995). 7612397 10.1006/jsbi.1995.1013

[b15] Humbel B. M. . *In situ* localization of beta-glucans in the cell wall of Schizosaccharomyces pombe. Yeast 18, 433–444 (2001). 11255251 10.1002/yea.694

[b16] Feofilova E. P. The fungal cell wall: modern concepts of its composition and biological function. Microbiology 79, 711–720 (2010). 21774151

[b17] Tougan T., Chiba Y., Kakihara Y., Hirata A. & Nojima H. Meu10 is required for spore wall maturation in Schizosaccharomyces pombe. Genes to Cells 7, 217–231 (2002). 11895484 10.1046/j.1356-9597.2001.00511.x

[b18] Huang Y. S., Karashima T., Yamamoto M. & Hamaguchi H. Molecular-level pursuit of yeast mitosis by time- and space-resolved Raman spectroscopy. Journal of Raman Spectroscopy 34, 1–3 (2003).

[b19] Huang Y. S., Karashima T., Yamamoto M., Ogura T. & Hamaguchi H. Raman spectroscopic signature of life in a living yeast cell. Journal of Raman Spectroscopy 35, 525-+ (2004).

[b20] Manners D. J. & Meyer M. T. The molecular structures of some glucans from the cell walls of Schizosaccharomyces pombe. Carbohydrate Research 57, 189–203 (1977).

[b21] Noothalapati H. & Shigeto S. Exploring metabolic pathways *in vivo* by a combined approach of mixed stable isotope-labeled Raman microspectroscopy and multivariate curve resolution analysis. Anal Chem 86, 7828–7834 (2014). 24975289 10.1021/ac501735c

[b22] Noothalapati Venkata H. N. & Shigeto S. Stable isotope-labeled Raman imaging reveals dynamic proteome localization to lipid droplets in single fission yeast cells. Chem Biol 19, 1373–1380 (2012). 23177192 10.1016/j.chembiol.2012.08.020

[b23] Huang Y. S., Karashima T., Yamamoto M. & Hamaguchi H. Molecular-level investigation of the structure, transformation, and bioactivity of single living fission yeast cells by time- and space-resolved Raman spectroscopy. Biochemistry 44, 10009–10019 (2005). 16042377 10.1021/bi050179w

[b24] Corbett E. C., Zichy V., Goral J. & Passingham C. Fourier transform Raman studies of materials and compounds of biological importance-II. The effect of moisture on the molecular structure of the alpha and beta anomers of d-glucose. Spectrochimica Acta Part A: Molecular Spectroscopy 47, 1399–1411 (1991).

[b25] Cael J. J., Koenig J. L. & Blackwell J. Infrared and raman spectroscopy of carbohydrates : Part IV. Identification of configuration- and conformation-sensitive modes for D-glucose by normal coordinate analysis. Carbohydrate Research 32, 79–91 (1974).

[b26] Synytsya A. . Glucans from fruit bodies of cultivated mushrooms Pleurotus ostreatus and Pleurotus eryngii: Structure and potential prebiotic activity. Carbohydrate Polymers 76, 548–556 (2009).

[b27] Mikkelsen M. S. . Comparative spectroscopic and rheological studies on crude and purified soluble barley and oat *β*-glucan preparations. Food Research International 43, 2417–2424 (2010).

[b28] de Medina-Redondo M. . The beta-1,3-glucanosyltransferase gas4p is essential for ascospore wall maturation and spore viability in Schizosaccharomyces pombe. Mol Microbiol 68, 1283–1299 (2008). 18410286 10.1111/j.1365-2958.2008.06233.x

[b29] Garcia I., Tajadura V., Martin V., Toda T. & Sanchez Y. Synthesis of alpha-glucans in fission yeast spores is carried out by three alpha-glucan synthase paralogues, Mok12p, Mok13p and Mok14p. Mol Microbiol 59, 836–853 (2006). 16420355 10.1111/j.1365-2958.2005.04995.x

[b30] Gendrin C., Roggo Y. & Collet C. Pharmaceutical applications of vibrational chemical imaging and chemometrics: A review. Journal of Pharmaceutical and Biomedical Analysis 48, 533–553 (2008). 18819769 10.1016/j.jpba.2008.08.014

[b31] Miljkovic M. . Label-free imaging of human cells: algorithms for image reconstruction of Raman hyperspectral datasets. Analyst 135, 2002–2013 (2010). 20526496 10.1039/c0an00042f

[b32] Sasic S. An in-depth analysis of Raman and near-infrared chemical images of common pharmaceutical tablets. Appl Spectrosc 61, 239–250 (2007). 17389063 10.1366/000370207780220769

[b33] Ando M. & Hamaguchi H. Molecular component distribution imaging of living cells by multivariate curve resolution analysis of space-resolved Raman spectra. J Biomed Opt 19, 011016 (2014). 24108582 10.1117/1.JBO.19.1.011016

[b34] Huang C. K., Ando M., Hamaguchi H. O. & Shigeto S. Disentangling dynamic changes of multiple cellular components during the yeast cell cycle by *in vivo* multivariate Raman imaging. Anal Chem 84, 5661–5668 (2012). 22686107 10.1021/ac300834f

[b35] Lichko L., Kulakovskaya T., Pestov N. & Kulaev I. Inorganic polyphosphates and exopolyphosphatases in cell compartments of the yeast Saccharomyces cerevisiae under inactivation of PPX1 and PPN1 genes. Biosci Rep 26, 45–54 (2006). 16779667 10.1007/s10540-006-9003-2

[b36] Jakubowski H. Sporulation of the yeast Saccharomyces cerevisiae is accompanied by synthesis of adenosine 5′-tetraphosphate and adenosine 5′-pentaphosphate. Proceedings of the National Academy of Sciences 83, 2378–2382 (1986). 10.1073/pnas.83.8.2378 PMC323300 3517867

[b37] Wright W. F., Overman S. B. & Ribes J. A. (1–3)-beta-D-Glucan Assay: A Review of its Laboratory and Clinical Application. Labmedicine 42, 679–685 (2011).

[b38] Fontana C. . (1–3)-beta-D-Glucan vs Galactomannan Antigen in Diagnosing Invasive Fungal Infections (IFIs). Open Microbiol J 6, 70–73 (2012). 22942923 10.2174/1874285801206010070 PMC3431562

[b39] Sulahian A. . Use and limits of (1–3)-beta-d-glucan assay (Fungitell), compared to galactomannan determination (Platelia Aspergillus), for diagnosis of invasive aspergillosis. J Clin Microbiol 52, 2328–2333 (2014). 24740084 10.1128/JCM.03567-13 PMC4097729

[b40] Lamoth F. . beta-Glucan antigenemia assay for the diagnosis of invasive fungal infections in patients with hematological malignancies: a systematic review and meta-analysis of cohort studies from the Third European Conference on Infections in Leukemia (ECIL-3). Clin Infect Dis 54, 633–643 (2012). 22198786 10.1093/cid/cir897

