# Supplementary material for: Label-free Chemical Imaging of Fungal Spore Walls by Raman Microscopy and Multivariate Curve Resolution Analysis
Source: Sci Rep. 2016 Jun 9;6:27789. doi: 10.1038/srep27789 (PMC4899791; doi:10.1038/srep27789)
Supplement: Supplementary Information [file srep27789-s1.pdf]

Label-free Chemical Imaging of Fungal Spore Walls by Raman Microscopy and Multivariate Curve Resolution Analysis

Hemanth Noothalapati[1,*], Takahiro Sasaki[1], Tomohiro Kaino[1,2], Makoto Kawamukai[1,2], Masahiro Ando[3], Hiro-o Hamaguchi[3,4] and Tatsuyuki Yamamoto [1,2,*]

[1] Raman Center for Medical and Biological Applications, Shimane University, Matsue 690-8504, Japan; [2] Faculty of Life and Environmental Science, Shimane University, Matsue 690-8504, Japan; [3] Consolidated Research Institute for Advanced Science and Medical Care, Waseda University, Tokyo 162-0041, Japan; [4] Institute of Molecular Science and Department of Applied Chemistry, National Chiao Tung University, Hsinchu 30010, Taiwan

[*] To whom correspondence may be addressed.

Dr. Hemanth Noothalapati
Raman Center for Medical and Biological Applications
Shimane University
1060, Nishikawatsu Cho, Matsue 690-8504, Shimane, Japan
Phone: +81-852-32-6571
Email: nvhnag@life.shimane-u.ac.jp

Or

Prof. Tatsuyuki Yamamoto
Faculty of Life and Environmental Science
Shimane University
1060, Nishikawatsu Cho, Matsue 690-8504, Shimane, Japan
Phone: +81-852-32-6551
Email: tyamamot@life.shimane-u.ac.jp

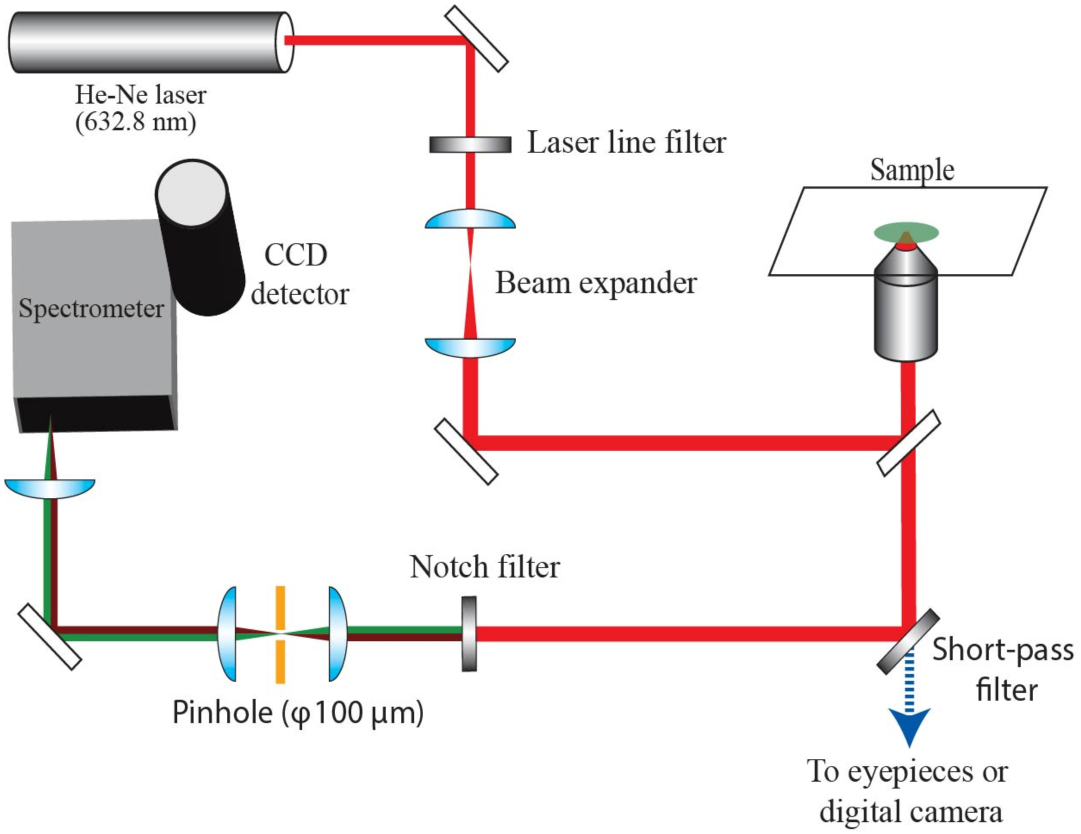

Supplementary Figure 1: Optical layout of Raman microscope used.

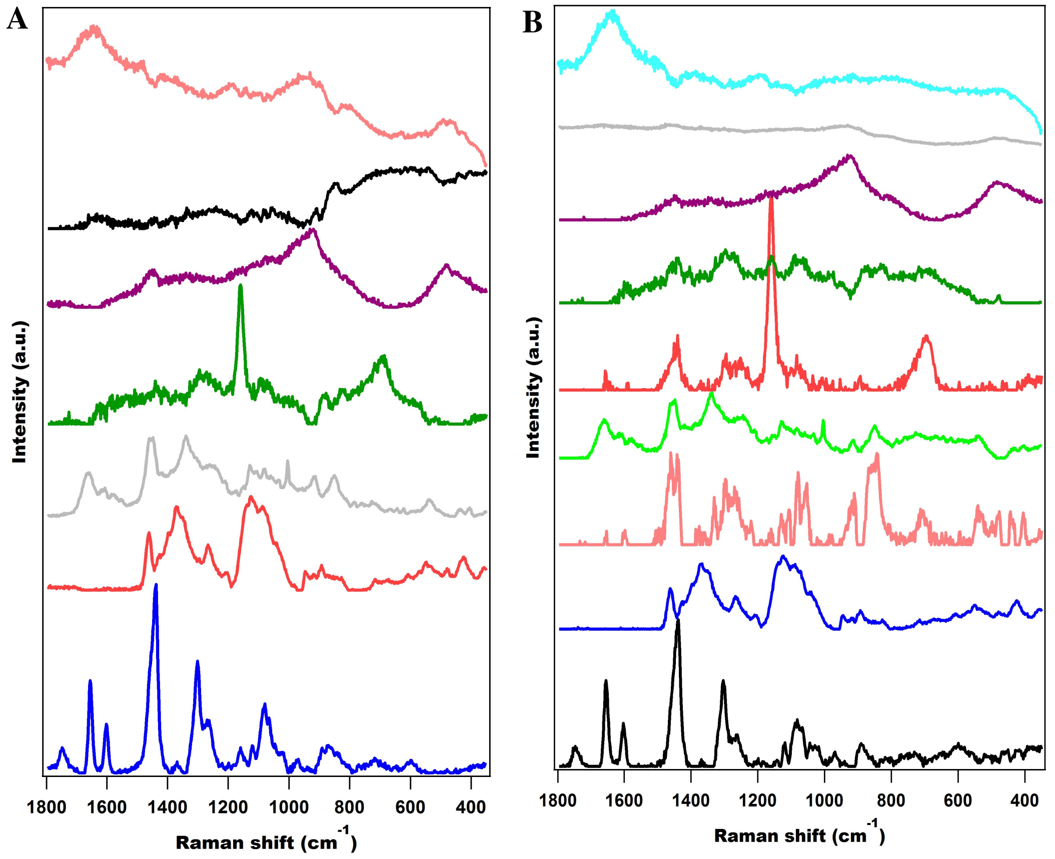

Supplementary Figure 2: MCR analyzed Raman spectra from **A)** 7 components model and **B)** 9 components model.

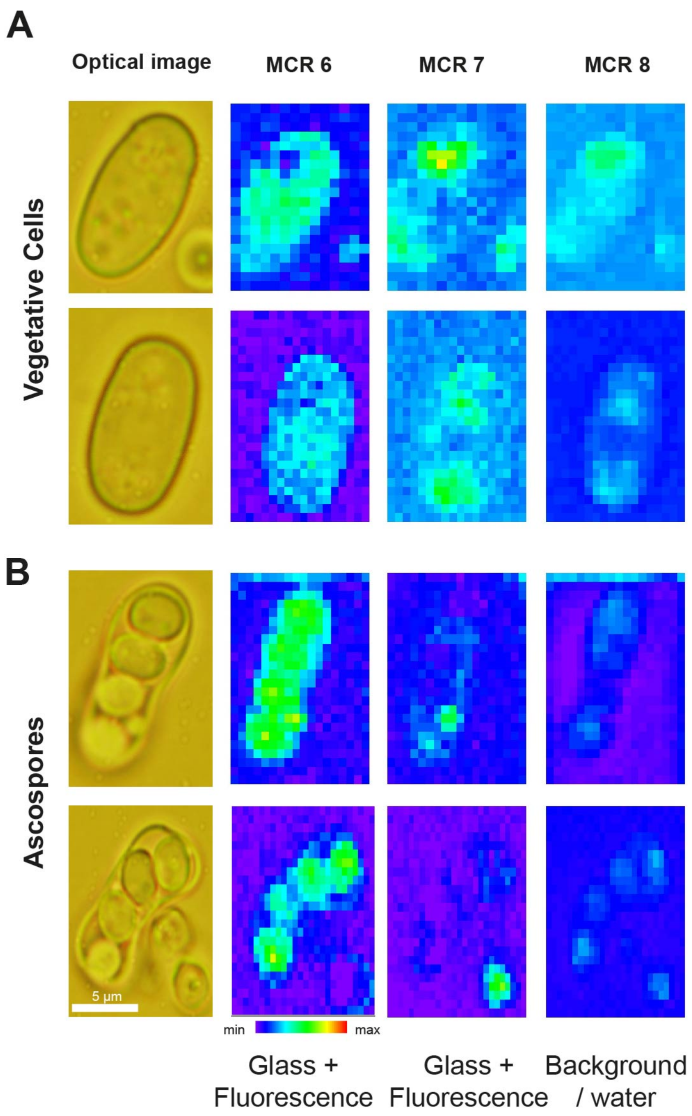

Supplementary Figure 3: Raman images constructed from MCR analysis from last three components shown in the main text. **A**) *S. pombe* vegetative cells and **B**) ascospores.

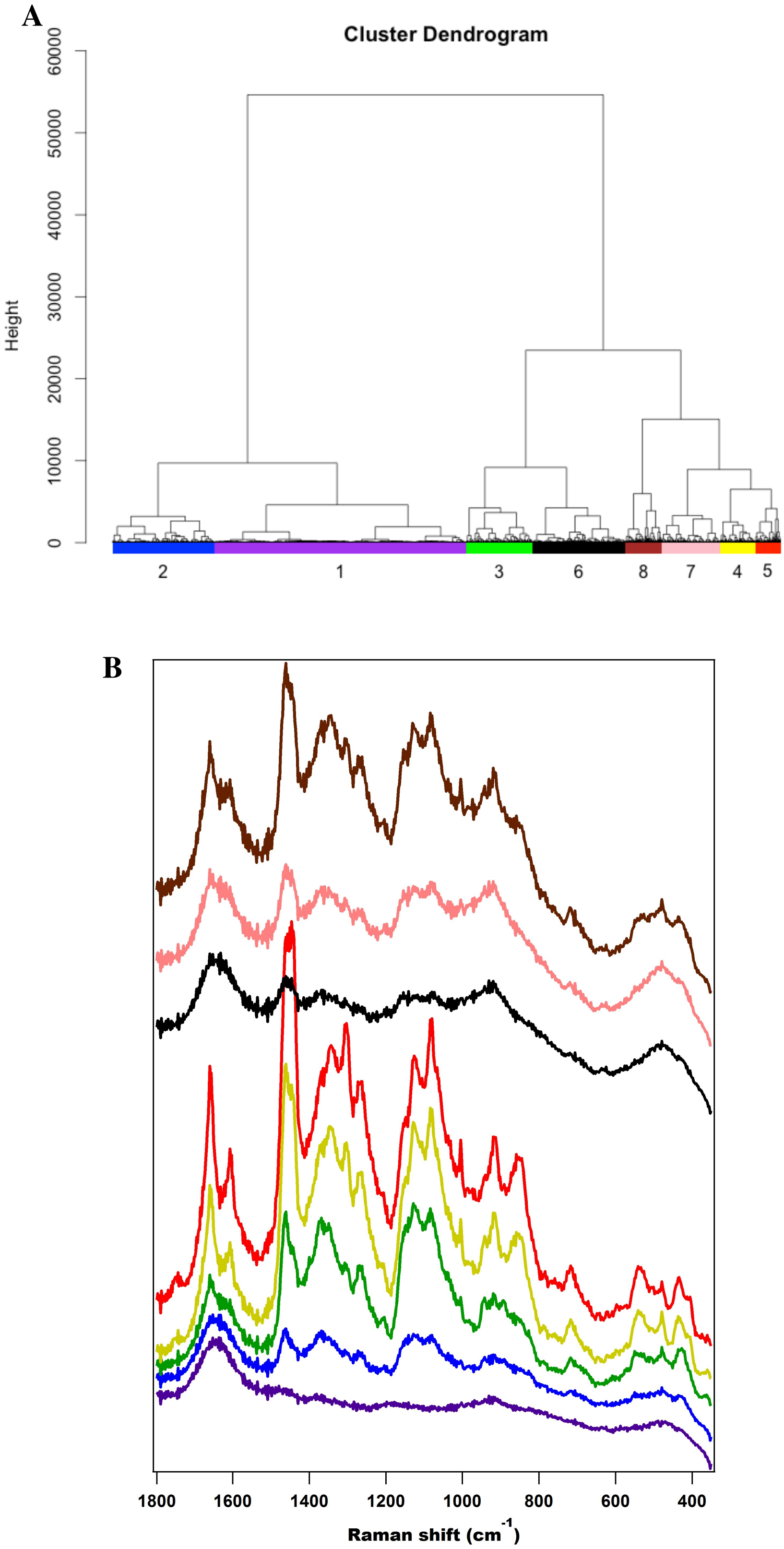

Supplementary Figure 4: 8 components AHCA. **A)** Dendrogram and **B)** Cluster spectra.
